# Practical Notes and Formulæ

**Published:** 1880-06-20

**Authors:** 


### PRACTICAL NOTES AND FORMULÆ.

**Cinchonia Alkaloid.**——This tasteless alkaloid of the bark, prepared by Messrs. Powers & Weightman, we have found to be a valuable agent. The absence of taste makes it very convenient for children and those of delicate stomachs, to whom the sulphate of quinine is so objectionable on account of its extreme bitterness.

As an antiperiodic it is reliable and efficient, and for the poor is the more acceptable on account of its cheapness, being only about one-sixth that of quinia; and in consequence of this fact, very favorably received by a great many practitioners, who are forced to donate to the indigent no small quantity of medicine.

In bilious remittent fever or intermittent fever we like the following method of using it, especially if the case be one in the country which we cannot see often, and in which there is doubt as to the exact chill time:

℞. (For an adult.)
Cinchonia alkaloid....................grs. xxiv.
Ft. powders No. viij.

Take one every three hours continuously until all are taken, using a little lemonade as a drink. If the patient be a female, lessen the dose. Or if a child, give one to two grains in syrup in the same manner.

For menorrhagia, we have found it useful in connection with elixir vitriol as a drink; best adapted to cases of relaxed or debilitated habit, and in cases having malarial complication.

It seems to act by toning up the capillaries and equalizing the circulation.

In dysmenorrhea and neuralgic affections connected with uterine disorders, it is useful for the same reason. In these cases it is best combined with anodynes, as follows:

℞. Cinchonia alkaloid..................grs. xxiv.
Pulvis doveri........................grs. xv.
M. Ft. powders No. vi.

Take one three times a day.

**Worms in the Nasal Cavities Expelled by Means of Chloroform.**—A woman twenty-nine years of age, was attacked by variola in the ninth month of pregnancy; normal accouchement occurred on the second day of the eruption, the disease following its regular course. During the period of desquamation, a fly entered the nasal cavity and deposited there its eggs, which were soon transformed into larvæ. Fever, violent cephalalgia, and multiplication of the larvæ followed. Insufflations of calomel and injections of salt water were useless. Dr. Garuco then had recourse to inhalations of chloroform, and at the first trial, seventy larvæ were expelled. This treatment was repeated every day, and completely relieved the patient.

Experiments with some of the larvæ showed that, at first, chloroform caused very active movements, after which all movements ceased and complete inertia ensued.—*La France Medicale.*

**Veratrum in Dysentery.**—In acute dysentery of threatening character, I have called in the aid of this agent with a prompt and unerring reduction of the inflammation. In one case, where the febrile movement was of a sthenic character, the patient, by mistake or restlessness, took a poisonous dose of it about twelve hours after I commenced its administration, and after that had not another dysenteric movement, and I feared for a little while would never have another. Opium, brandy and a dry heat restored her. I have had several patients take hypermedicinal doses, and get a sudden jugulation (as the French say) of their pneumonia or other inflammation.

In good, honest, acute articular rheumatism—the more severe the better—I regard veratrum as serviceable as in pneumonia. This, with opium and sal Rochelle, will do the work quicker than salicylic acid. Veratrum as a sedative, sal Rochelle as an alkaline element, and opium to subdue pain and restrain over-action of the salt, and there we have the thing in a nutshell.—DR. GREEN *in Med. and Surg. Rep.*

**Vermifuge.**—

| | | |
|---|---|---|
| ℞ | Santonine................................ | grs. vi. |
| | Podophyllin.............................. | grs. j. |
| | Sach. lacta.............................. | grs. xxx. |

M. and triturate thoroughly. Sig. Give each day grs. ij., morning, noon and night, in sweetened water.

The above "worm powders" secured the dislodgment and expulsion, per rectum, of sixteen ascaris lumbricoides averaging ten inches in length, and to the great astonishment of the friends of the little sufferer as well as myself, he passed a section of tænia solium twelve feet in length.—*Eclectic Medical Journal.*

**Tartrate of Morphia.**—This new preparation is recommended as being very soluble, and passing quickly out of the system, leaving less unpleasant after effects than either the muriate or acetate. Its great solubility makes it particularly advantageous for subcutaneous injection. It gives little smarting or irritation when thus administered, and the solution never clogs the finest needles.—*Louisville Medical News.*

**For Dysentery.**—Carlsbad salts will be found a good remedy in the early stages, or epsom salts, as follows:

| | | | |
|---|---|---|---|
| ℞ | Sulphate of magnesia...................... | ʒ i. | |
| | Morphine.................................. | gr. ¼. | |
| | Water..................................... | ℥ iv. | |
| | Spirits of camphor........................ | gtt. x. | M. |

Take one teaspoonful every one to two hours.

**An Emetic for Infants.**—Dr. S. W. Smith writes:

I beg leave to record that half a teaspoonful of glycerine acts as a simple and efficient emetic for infants. Perhaps some of your readers can confirm this by future experience.—*British Med. Journal.*

**Inflammation of the Middle Ear.**—Bezölds recommends as antiseptic measures in inflammation of the middle ear :

1. Disinfection of the meatus and of the cavity of the drum by means of a four-per-cent. solution of boracic acid.
2. Insufflation of finely-powdered boracic acid.
3. Closure of the meatus with salicylated or borated lint or cotton.

The ahove treatment was carried out in twenty-nine acute and one hundred and two chronic cases, with unvarying success. The time occupied in producing a cure was considerably less than by other methods. In the destructive necroses of bone which had developed in advanced cases of phthisis pulmonalis, and the suppurative inflammations of the roof of the drum cavity, which ended in rupture of the membrana shrapnelli, or passed to the formation of polypi at their site, this treatment was not so successful.—*Med. Herald.*

**For Fresh Cold in the Head.**—Dr. T. F. Houston writes: For fresh cold in the head, accompanied with obstruction in the nasal passages:

| | | | |
|---|---|---|---|
| ℞ | Carbolic acid.............................. | ʒ i. | |
| | Absolute alcohol.......................... | ʒ ii. | |
| | Caustic solution of ammonia.............. | ʒ i. | |
| | Aqua distillat............................ | ʒ iii. | M. |

Make a cone of writing paper, put a small piece of cotton in it, drop on the cotton ten drops of the mixture, and inhale until all is evaporated. Repeat this every two hours until relieved.

**Oleate of Lead in Eczema.**—Mr. J. Sawyer, of London, gives in the Practitioner the following formula:

| | |
|---|---|
| Lead oleate........................... | 24 parts. |
| Heavy and inodorous paraffin oil..... | 14 parts. |

The lead oleate is prepared by heating a mixture of oleic acid and oxide of lead. He can confidently recommend this ointment as a very efficient local application in eczema. He has used it successfully in a large number of cases.—*Med. and Surg. Reporter.*

**Simple Mode of Reducing a Paraphimosis.**—Where ordinary means fail, Bordinet proceeds as follows: He takes a hair-pin, presses the points together somewhat, and inserts the curved end under the strangulation back of ihe glans. He then applies a second and a third at intervals around the gians; then, drawing the prepuce forward, reduces it with great facility, the skin sliding over the three bridges without obstruction.—*Chicago Med. Jour. and Examiner.*

**Richardson's Styptic Colloid.**—

| | | | |
|---|---|---|---|
| ℞ | Acid tannici................................ | ℥ ii. | |
| | Alcoholis absoluti.......................... | f. ℥ ss. | |
| | Etheris.................................... | ℥ iiss. | |
| | Collodion, qs. ad.......................... | ℥ xii. | M. |

—*Monthly Review of Medicine and Pharmacy.*

